# Intrastromal Corneal Ring Segment Implantation in a Patient with the Bowman Layer Inlay Transplantation for Keratoconus

**DOI:** 10.1155/2023/9967223

**Published:** 2023-05-22

**Authors:** João Ponces Ramalhão, Miguel Mesquita Neves, Miguel Gomes, Luís Oliveira

**Affiliations:** Ophthalmology Department, Centro Hospitalar do Porto Largo Professor Abel Salazar, 4099-001 Porto, Portugal

## Abstract

Keratoconus (KC) is a bilateral ectatic corneal disease which results in changes in the corneal architecture and can lead to severe visual impairment. Treatment options depend on the stage of the disease, and they aim either at improving vision or arrest progression. The Bowman layer transplantation (BLT) is a recent surgical option in patients with KC and may postpone corneal transplantation in some patients. We present a case of a 22-year-old patient with a 10-year follow-up history of progressing KC. A first attempt for an intracorneal ring segment (ICRS) implantation when he was 13 years old was unsuccessful due to a superficially implanted segment. At that time, collagen cross-linking was unavailable, and his young age raised concerns about performing a penetrating keratoplasty/lamellar keratoplasty. A BLT was performed with further ICRS implantation with relative disease stability and visual improvement. ICRS implantation in KC patients with BLT has not previously been described in literature and can be an option in selected patients.

## 1. Introduction

KC is a bilateral ectatic corneal disease, generally asymmetric, with an established prevalence of 1/2000 within the general population [1, 2]. It results from changes in the organization of the stromal lamellae with an unequal distribution of collagen fibers, with a reduction of the number of keratocytes, particularly around the apex of the cone [3].

Advances in diagnostic and staging exams such as corneal tomography have allowed for an early diagnosis of this patient with mild to moderate disease, for whom different strategies can be adopted [4, 5]. These strategies range from spectacles and hard contact lenses to various innovative surgical procedures. In advanced cases, a penetrating keratoplasty (PK) and a deep anterior lamellar keratoplasty (DALK) are the only options recommended, although associated to a considerable high risk of intraoperative and postoperative complications. In order to delay the need for these techniques, corneal collagen cross-linking (CXL) and intrastromal corneal ring segments (ICRS) have been used in the past decades in patients with KC [6, 7]. CXL is generally indicated for progressing KC with at least 400 *μ*m corneal thickness, and its effectiveness is higher in corneas with Kmax under 58D. ICRS implantation is indicated for patients with KC with Kmax <60D aiming for better vision [8].

More recently, the Bowman layer transplantation (BLT) was introduced as a new option for the KC treatment. In 2014, Dragnea et al. and van Dijk et al. were the first to describe the utility of BLT in patients with advanced progressive KC. It is indicated for the eyes with progressing advanced keratoconus who are not eligible for CXL or ICRS due to an extremely thin or steep cornea [7, 8]. Nevertheless, once a BLT is performed and the pathologic steepening is flattened, the combination with an ICRS can be a possibility to optimize even further visual acuity and contact lens adaptation.

## 2. Case Report

**Figure 1:**
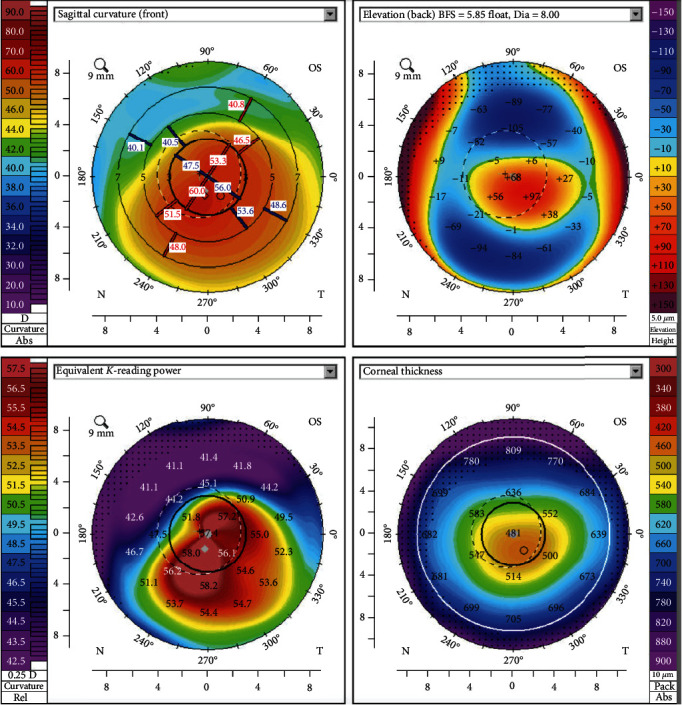
OS tomographic maps at first appointment.

We present a case of a 22-year-old male with a bilateral and highly asymmetric KC diagnosed when he was 12 years old. His best corrected visual acuity (BCVA) at that time was 20/20 (-0.50-1.00 × 30) right eye (OD) and 20/80 (-3.50-5.00 × 145) left eye (OS). OS corneal tomography revealed a flat meridian (K1) of 51.7D, a steep meridian (K2) of 56.3D, maximum keratometry (Kmax) of 63.3D, and thinnest corneal thickness (TCT) of 455 *μ*m (shown in Figure 1).

At that time, collagen cross-linking was unavailable at our ophthalmology department and an OS manual ICRS implantation was done. Two months later, the ICRS was explanted due to a corneal neovascularization associated with a superficially implanted ICRS. Although he had an advanced KC, corneal transplantation was delayed due to his young age and the patient had a follow-up with close surveillance.

During the following 7 years, his BCVA OS declined to 20/200 (-3.50-5.00 × 145) and his OS tomographic parameters progressed to K1 of 52.9D, K2 of 58.1D, Kmax 64.3D, and TCT of 454 *μ*m. Contact lens adaptation was not successful for this patient as he could not tolerate them.

**Figure 2:**
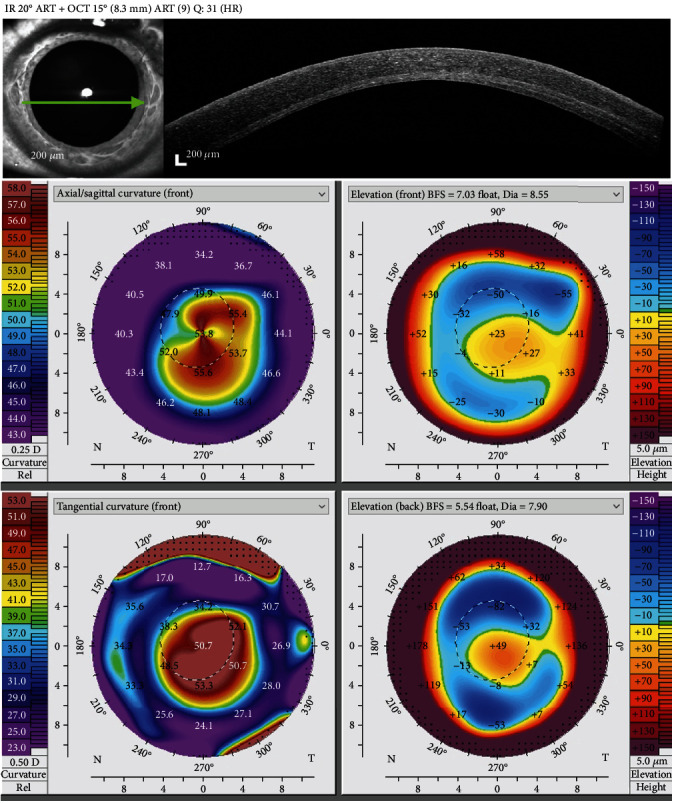
OS cornea OCT and OS tomographic maps two months after BLIT.

When he became 19 years old, an uneventful Bowman layer inlay transplantation (BLIT) was performed. Two months after surgery, BCVA improved to 20/80 (-3.50-5.00 × 145). Corneal tomography showed an improvement in corneal steepening and corneal thickness (K1 51.2D, K2 54.7D, Kmax 57.4D, and TCT 456 *μ*m) (shown in Figure 2).

**Figure 3:**
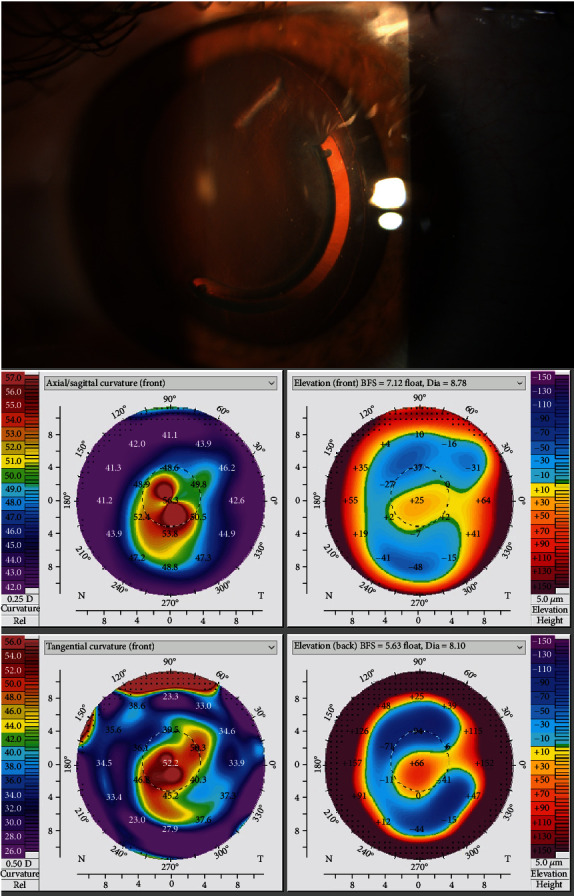
ICRS implantation after BLIT (with its visible interface) and OS tomographic maps after BLIT and FS-ICRS implantation.

During the following 3 years, a worsening in corneal curvatures (K1 51.7D, K2 57.1D, and Kmax 62.4D) was noted despite of the visual acuity which remained stable. By the time he was 22 years old, in order to further improve his visual acuity and due to the amelioration in the patient's corneal tomography after BLIT (which would consequently improve the prognosis associated with a new ICRS implantation), a decision was made to perform again an ICRS implantation. An uneventful femtosecond-assisted ICRS (FS-ICRS) implantation (Keraring 5 mm segment AS 160/200-300 W clockwise, with a 60° incision and 400 *μ*m depth) was done, according to the normogram proposed by Mediphacos. One year after the procedure, the patient's BCVA became 20/40 (-1.00-5.00 × 130) and corneal tomography parameters were K1 45.4D, K2 51.2D, Kmax 59.0D, and TCT 509 *μ*m (shown in Figure 3).

During all this period, his right eye (OD) was only minimally affected, with a BCVA that declined from 20/20 (-0.50-1 × 30) to 20/25 (−1.00 × 90). No interventions OD were yet required.

## 3. Discussion

The Bowman layer plays a major role in the corneal biomechanics as it is the second strongest element, after the anterior third of the stroma [9]. Its fragmentation is a pathognomonic feature of advanced KC [10, 11]. BLIT procedure consists of a midstromal implant of a donor-isolated Bowman layer for patients with advanced KC, especially in patients not eligible for CXL, postponing the need for DALK or PK [7, 12].

Once BLT is performed, a significative flattening of the cone can allow for a previously contraindicated ICRS implantation to be allowed. In theory, ICRS implantation in a cornea previously submitted to a BLT would not only flatten even further the cornea (allowing for a better CL adaptation) but would also be associated with an improvement in irregular astigmatism and higher order aberrations like coma, improving the patient vision [6].

In this case, the patient has a KC since he was 12 years old. Its disease is substantially asymmetrical as his right eye remained with good BCVA throughout all follow-up time, and his left eye developed an advanced KC. His OS BCVA at presentation was 20/80, and his corneal tomography at that time showed parameters which allowed the implantation of an ICRS, which was not successful due to a superficially implanted segment. Contact lens adaptation was not successful for this patient as he could not tolerate them. Subsequently, his disease progressed, and CXL was unavailable at that time in our center. If available, it would have been the best option to arrest disease progression, even in combination with ICRS [13, 14]. An annual surveillance regimen was adopted, and corneal transplantation (DALK or PK) was delayed as the patient was very young. With the introduction of BLT in surgical practice, this patient became a good candidate for this technique and BLIT was performed by the time he was 19 years old. After the intervention, BCVA improved from 20/200 to 20/80 and his corneal tomographic parameters also significantly improved. With the improvement of his corneal topography due to the BLIT and disease stability, the decision to make a second attempt for an ICRS was done. Therefore, when he was 22 years old, a single ICRS was implanted which allowed further improvement in his BCVA, reaching 20/40 with a further improvement in his keratometry values.

This case shows good clinical and tomographic results after an ICRS implantation in a patient with an advanced KC and a previous BLIT. Without BLIT and in the absence of CXL at our center at that time, we believe the most likely outcome for this patient would have been the need for a lamellar or penetrating keratoplasty. However, BLIT was able to slow the progression of KC and improve our patient's visual acuity due to a corneal surface regularization. Furthermore, it prevented the need for an immediate DALK or PK. As a relatively stable disease was achieved, an ICRS was performed to further improve functional outcome. ICRS implantation is also an option in this subset of patients, and it is our purpose to report its efficacy in this case. To our best knowledge, implantation of ICRS in a patient who has been previously submitted to a BLIT has not yet been described. We believe that this case may demonstrate the efficacy of that option. Nevertheless, new studies including more similar cases are required to confirm the effectiveness of this approach as this is an isolated case and the follow-up after ICRS implantation is only one year.

## Data Availability

All data generated or analyzed during this study are included in this article. Further enquiries can be directed to the corresponding author.
